# Impregnation of bone chips with antibiotics and storage of antibiotics at different temperatures: an *in vitro *study

**DOI:** 10.1186/1471-2474-11-96

**Published:** 2010-05-25

**Authors:** Nina MC Mathijssen, Pieter LC Petit, Peter Pilot, B Wim Schreurs, Pieter Buma, Rolf M Bloem

**Affiliations:** 1 Netherlands Bone bank Foundation, Rijnsburgerweg 10, 2333 AA Leiden, The Netherlands; 2 Departement of Microbiology, Vlietland Hospital, Vlietlandplein 2, 3118 JH Schiedam, The Netherlands; 3 Department of Orthopaedics, Reinier de Graaf Gasthuis, Reinier de Graafweg 3/11, 2625 AD Delft, The Netherlands; 4 Department of Orthopaedics, UMC St. Radboud, Theodoor Craanenlaan 7, 6525 GH Nijmegen, The Netherlands; 5 Orthopaedic Research Laboratory, Department of Orthopaedics, UMC St. Radboud, Theodoor Craanenlaan 7, 6525 GH Nijmegen, The Netherlands

## Abstract

**Background:**

Allograft bone used in joint replacement surgery can additionally serve as a carrier for antibiotics and serve as a prophylaxis against infections. However, *in vitro *dose-response curves for bone chips impregnated with different kinds of antibiotics are not available. In addition, while it would be desirable to add the antibiotics to allograft bone chips before these are stored in a bone bank, the effects of different storage temperatures on antibiotics are unknown.

**Methods:**

Five different antibiotics (cefazolin, clindamycin, linezolid, oxacillin, vancomycin) were stored, both as pills and as solutions, at -80°C, -20°C, 4°C, 20°C and 37°C; in addition, bone chips impregnated with cefazolin and vancomycin were stored at -80°C and -20°C. After 1 month, 6 months and 1 year, the activity of the antibiotics against *Staphylococcus epidermidis *was measured using an inoculated agar. The diameter of the *S. epidermidis*-free zone was taken as a measure of antibiotic activity.

In a separate experiment, *in vitro *dose-response curves were established for bone chips impregnated with cefazolin and vancomycin solutions at five different concentrations.

Finally, the maximum absorbed amounts of cefazolin and vancomycin were established by impregnating 1 g of bone chips with 5 ml of antibiotic solution.

**Results:**

A decrease of the *S. epidermidis*-free zone was seen with oxacillin and cefazolin solutions stored at 37°C for 1 month, with vancomycin stored at 37°C for 6 months and with cefazolin and oxacillin solutions stored at 20°C for 6 months. The activity of the other antibiotic solutions, pills and impregnated bone chips was not affected by storage. The *in vitro *dose-response curves show that the free-zone diameter increases logarithmically with antibiotic concentration. The absorbed antibiotic amount of one gram bone chips was determined.

**Conclusions:**

Storage of antibiotics in frozen form or storage of antibiotic pills at temperatures up to 37°C for 12 months does not affect their activity. However, storage of antibiotic solutions at temperatures above 20°C does affect the activity of some of the antibiotics investigated. The *in vitro *dose-response curve can be used to determine the optimal concentration(s) for local application. It provides the opportunity to determine the antibiotic content of bone chips, and thus the amount of antibiotics available locally after application.

## Background

Infection is one of the most devastating adverse events following joint replacement surgery. Deep infection rates in primary hip replacement surgery are around 0.5% to 2%. These infections result in a reduced quality of life [1,2]. The patient often needs several re-operations, and if surgical debridement is not sufficient a Girdlestone situation is created; the patient then has to function without prosthesis for three to six months, which markedly limits physical activity. Furthermore, the hospitalisation costs for an infected patient are about 3.7 times those for a similar, uninfected patient [1].

Bone impaction grafting (BIG) for joint replacement surgery has been used with satisfactory results in clinical practice, for acetabular reconstructions since 1979 and for femoral reconstructions since 1987 [3-10]. With BIG, first the segmental bone defects in acetabulum and femur are restored with metal meshes. Next, these defects are filled with tightly impacted morselized cancellous bone chips in combination with a cemented prosthesis. Osteoclasts will resorb necrotic graft remnants and osteoblasts will form new woven bone. This remodelling of newly formed bone into its characteristic structure will lead to the biological repair of the defect following surgery [11].

Surgery with BIG is more complex and time-consuming than primary hip surgery which may contribute to a higher infection rate in hip revision surgery (percentages ranging from 2.0% to 2.5% [12-14]). BIG creates an avascular area where local circulation is disrupted. If infections arises, this may prevent antibiotics that are administered systemically to reach the infected bone [15]. In addition, the formation of a biofilm on the surface of the implant renders systemic antibiotics less effective [16].

Bone cements containing antibiotics were developed to solve this problem. These cements may serve as a drug delivery system and prophylaxis against infections as they make it possible to achieve higher local drug concentrations. However, controversy exists on the efficacy of the antibiotic-containing cements [17]. Probably 90% of the antibiotics contained in the cement is never released [18]. Only when cracks are formed in the cement layer will a small, sub-inhibitory amount of antibiotics be released into the surrounding tissue. This release can continue for years, potentially inducing resistance [18].

Several studies have shown that morselized allograft bone, which is used not only in BIG but also in other surgeries, can be made to act as a carrier for antibiotics, either by impregnating the bone grafts with various antibiotic solutions [19,23], or by mixing them with antibiotic powders [24,25]. Although these studies showed, *in vitro *as well as *in vivo*, that bone impregnated with antibiotics can be used effectively as prophylaxis against infections, they did not establish the concentration of antibiotic present in the bone after impregnation and how much was released locally. Furthermore, no correlation of antibiotic concentration and zone of inhibition was reported of the antibiotics used in combination with bone chips. Finally, allograft bone is stored at a bone bank at -80°C, then thawed in the operating room. In the impregnation studies, all the antibiotics were added to the thawed bone, immediately before use. It is thus unknown whether the antibiotics can be added before freezing without affecting their activity.

Based on this we investigated if storage of antibiotics and antibiotic-loaded bone grafts at different temperatures affects their activity. The correlation between antibiotic concentration and zones of inhibition for the bone/antibiotics composites was analysed. The results permit us to develop a standardized bone bank product in which the amount of antibiotics present on the bone chips, and therefore the amount of antibiotics released locally from the bone, will be known.

## Methods

### Measurement of activities

For all tests performed in this study, the following method was used:

Antibiotic pills, antimicrobial susceptibility test disks (Oxoid) with antibiotic solution dripped onto the disks and bone chips impregnated with antibiotic solution were placed on an inoculated iso agar (cefazolin, clindamycin, linezolid or vancomycin) or an inoculated Mueller Hinton agar (oxacillin). Bone chips were placed in a hole of 10 mm diameter punched into, but not through, the agar and then covered with 25 μl saline dripped onto them.

All agars were inoculated with 0.5 McFarland ATCC 12228 *Staphylococcus epidermidis *since this organism is one of the main pathogens colonizing biomaterials [26]. After storing the iso agars at 37°C and the Mueller Hinton agars at 30°C for 18-24 hours, the zones of inhibition were measured.

Antibiotic pills from the routine clinical lab were used as controls. The zone of inhibition of these pills is comparable to that of 10 μl antibiotic solution and that of chips freshly impregnated with 10 μl antibiotic solution.

Cefazolin, clindamycin, linezolid, oxacillin and vancomycin were used in this study, all antibiotics against gram stain positive micro-organisms, the main cause for orthopaedic infections. Oxacillin is the preferred treatment for systemic staphylococcal infections and in case of penicillin hypersensitivity the others are used (mainly cefazolin or clindamycin). For prophylaxis cefazolin or clindamycin are preferred and in case of infection with methicillin resistant Staphylococcus aureus (MRSA) vancomycin is used. In this way all important antibiotics against these gram stain positive micro-organisms were tested. The concentrations used for the tests are the concentrations used in the whole range of clinical situations: systemic or local infection and prophylactic use. In addition, high concentrations were used to determine local concentration limits.

Five different storage temperatures were used in the experiments. -80°C and -20°C are storage temperatures for allograft bone in bone banks. 4°C and 20°C are commonly used storage temperatures in the laboratory and 20°C is the temperature of the operation room. 37°C was included so as to investigate antibiotic stability at higher temperature and because this temperature is body temperature.

### Storage of antibiotic pills

Pills of five different antibiotics - cefazolin 60 μg, clindamycin 25 μg, linezolid 30 μg, oxacillin 1 μg and vancomycin 70 μg - were stored at five different temperatures. For each antibiotic, nine pills of each antibiotic were stored in a closed tube at each temperature. After 1 month, 6 months and 1 year of storage, three pills of each antibiotic were brought to room temperature and placed on an agar to determine the inhibition zone of the pills.

### Storage of antibiotic solutions

Five different antibiotic solutions - cefazolin 6.0 mg/ml, clindamycin 2.5 mg/ml, linezolid 3.0 mg/ml, oxacillin 0.1 mg/ml and vancomycin 7.0 mg/ml - were stored at five different temperatures. For each antibiotic solutions, 9 tubes of 1 ml, were stored at each temperature. After 1 month, 6 months and 1 year of storage, three tubes of each antibiotic solution were brought to room temperature and 0.10 μg of each antibiotic solution was dripped onto a antimicrobial susceptibility test disk which was then placed on an agar.

### Storage of bone chips impregnated with antibiotic solutions

**Figure 1:**
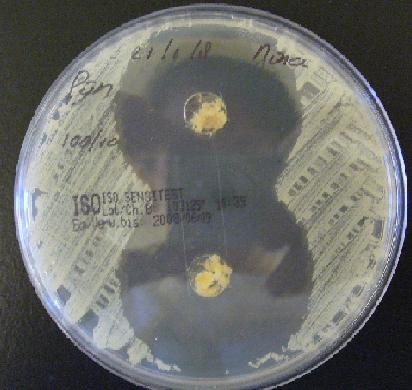
**Bone chips on an inoculated iso agar**.

60 tubes, each containing 0.10 g of bone chips from a human morselized femoral head, were impregnated with 60 μg/10 μl cefazolin solution for ten minutes, then 30 were stored at -80°C and 30 at -20°C. Similarly, 60 tubes with 0.10 g bone chips each were impregnated with 70 μg/10 μl vancomycin and 30 were stored at -80°C and 30 at -20°C. Other storage temperatures were not tested, since bone allografts cannot be used for transplantation after storage at temperatures above -20°C for more than one month. After 1 month and 6 months of storage, 20 tubes each of the cefazolin- and the vancomycin-impregnated bone chips (ten tubes from -80°C and 10 tubes from -20°C storage) were thawed and placed on iso agars (figure 1).

### Correlation of antibiotic concentration and zone of inhibition of bone impregnated with vancomycin and cefazolin

Five different cefazolin solutions and five different vancomycin solutions were used to impregnate bone chips from a morselized femoral head and to determine the correlation of antibiotic concentration and zone of inhibition. The concentrations were chosen so as to exceed the minimal inhibitory concentration, and similar to that of the control pills from the clinical routine lab (cefazolin 60 μg and vancomycin 70 μg). Cefazolin: 0.5 μg/10 μl, 2.5 μg/10 μl, 10 μg/10 μl, 60 μg/10 μl and 100 μg/10 μl; vancomycin: 0.5 μg/10 μl, 2.5 μg/10 μl, 10 μg/10 μl, 70 μg/10 μl and 100 μg/10 μl. For each concentration, ten 0.10 g bone chips were impregnated at room temperature for ten minutes and placed on agar as described above; the mean diameter of the free zone was measured, and the antibiotic quantity was correlated to the zone of inhibition.

### Determination of the maximum amount of antibiotics absorbed by bone chips

One gram of bone chips from a morselized femoral head was impregnated with 5 ml of cefazolin or vancomycin solution. The maximum absorbed amount was then calculated based on the correlation between antibiotic concentration and zone of inhibition determined earlier.

Chips were impregnated with four different cefazolin solutions (1000 μg/5 ml, 750 μg/5 ml, 500 μg/5 ml and 250 μg/5 ml) and with four different vancomycin solutions (5000 μg/5 ml, 2500 μg/5 ml, 1000 μg/5 ml and 250 μg/5 ml). After ten minutes of impregnation the remaining solution was poured off. 0.10 gram was taken off the impregnated 1 gram and placed on an inoculated agar; this was repeated five times. The zone of inhibition around the chips was measured. Matching this zone to a zone of inhibition in the correlation analysis performed earlier yielded the matching amount of antibiotics and thus the maximum amount of antibiotic that can be absorbed at this concentration. In a repeat test, after the antibiotic solution was poured off the bone chips were rinsed twice with 5.0 ml saline in order to remove any antibiotic solution remaining on the surface of the bone chips; thus, the real amount absorbed by the bone chips was calculated.

## Results

### Storage of antibiotics

No differences were seen, compared to controls, in the zone of inhibition for cefazolin, vancomycin, linezolid and clindamycin pills that had been stored at -80°C, -20°C, 4°C, 20°C and 37°C for 1 month, 6 months and 1 year. Also, no differences in response at any of these temperatures were found for clindamycin and linezolid solutions that had been stored for 12 months.

**Figure 2:**
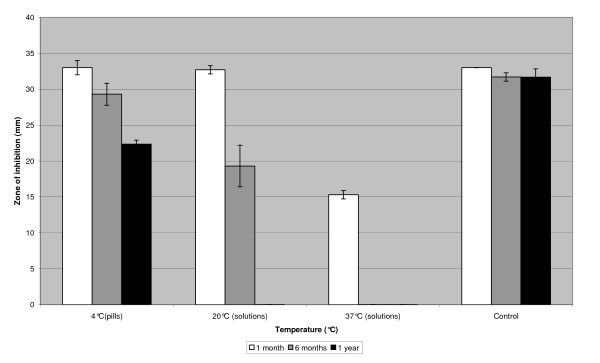
**Storage of oxacillin pills at 4°C and oxacillin solutions at 20°C and 37°C compared to controls**. Storage of oxacillin pills (1 μg) at 4°C and oxacillin solutions (0.1 mg/ml) at 20°C and 37°C for 1 month, 6 months and 12 months compared to controls.

Oxacillin pills stored at 4°C for 12 months showed a decreased zone of inhibition compared to controls. Oxacillin solutions showed a decreased zones of inhibition after 1 month of storage at 37°C and after 6 months of storage at 20°C. No zone of inhibition could be found after 12 months of storage at 20°C and after 6 months of storage at 37°C (figure 2). At other temperatures, no differences in the zone of inhibition were seen compared to controls.

**Figure 3:**
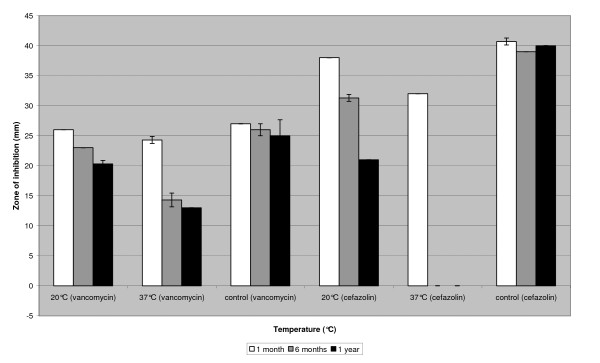
**Storage of vancomycin and cefazolin solutions at 20°C and 37°C compared to controls**. Storage of cefazolin solutions (6.0 mg/ml) and vancomycin solutions (7.0 mg/ml) at 20°C and 37°C for 1 month, 6 months and 12 months compared to controls.

Vancomycin solutions stored at 37°C for 6 months showed a decreased zone of inhibition, while the zone of inhibition was not affected after 1 month of storage at this temperature (figure 3). At -80°C, -20°C and 4°C no differences were visible compared to controls as was the case for vancomycin solutions stored at 20°C for 6 months; however, 12 months at 20°C did result in a decreased zone of inhibition (figure 3).

For cefazolin solutions, the zone of inhibition was decreased when compared to controls after storage at 37°C for 1 month and at 20°C for 6 and 12 months. No zone of inhibition could be seen after 6 and 12 months of storage at 37°C (figure 3).

### Storage of bone chips impregnated with antibiotics

**Figure 4:**
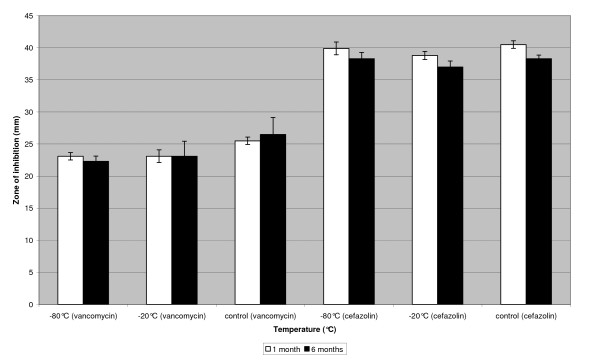
**Storage of bone chips impregnated with cefazolin and vancomycin solutions for 1 month and 6 months at -20°C and -80°C, compared to controls**. Storage of cefazolin solutions (6.0 mg/ml) and vancomycin solutions (7.0 mg/ml) at -80°C and -20°C for 1 month and 6 months compared to controls.

Bone chips impregnated with vancomycin or cefazolin, stored at -20°C and -80°C for 6 months did not show a difference in zone of inhibition when compared to controls (figure 4)

### Correlation of antibiotic concentration and zone of inhibition of bone chips impregnated with vancomycin and cefazolin

**Figure 5:**
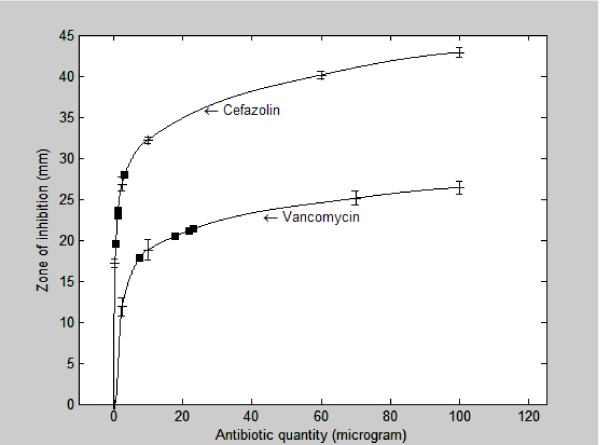
**Regression analysis of bone chips impregnated with antibiotic solutions**. Squares represent the inhibition zones for 0.10 g of bone chips taken from 1.0 g of bone chips impregnated with 5 ml of antibiotic solution and rinsed with saline twice; the fitted curves yield the corresponding antibiotic content.

The correlation of antibiotic quantity and zone of inhibition of bone chips impregnated with cefazolin and vancomycin is presented in figure 5. A curve was fitted between the quantity of antibiotic and diameter of inhibition zone measured. With increasing antibiotic quantity, the zone of inhibition increases logarithmically.

### Determination of the maximum absorbed amount

Table 1 represents the results of the impregnating bone chips with 5 ml cefazolin or vancomycin solution. The zones of inhibition of 0.10 g bone chips impregnated with 5 ml antibiotic solution (and rinsed with saline twice) are related to antibiotic quantity in figure 5 (represented by the squares). The associated quantity of antibiotic present in 0.10 g of chips can then be read from the figure, and from this, the antibiotic content of 1.0 g bone chips is determined (table 1).

**Table 1:** Impregnation of bone chips with cefazolin and vancomycin solutions

Antibiotic	Rinsing with 0.9% saline, +/-	Antibiotic content of impregnation fluid (μg/5 ml)	Mean diameter of *S. epidermidis*-free zone (mm)	Mean antibiotic content of 1.0 g of bone chips (μg)
Cefazolin	+	1000	28.0 ± 1.2	32

	+	750	23.6 ± 1.3	15

	+	500	23.0 ± 1.2	13

	+	250	19.6 ± 0.5	7

	-	1000	32.4 ± 0.9	105

	-	750	31.8 ± 0.8	92

	-	500	29.0 ± 2.4	44

	-	250	28.6 ± 0.5	39

Vancomycin	+	5000	23.2 ± 0.5	214

	+	2500	22.0 ± 1.0	212

	+	1000	17.8 ± 1.1	204

	+	250	7.6 ± 6.9	178

	-	5000	24.2 ± 0.8	215

	-	2500	23.6 ± 1.1	214

	-	1000	22.8 ± 3.3	212

	-	250	16.0 ± 0.7	201

Antibiotic quantity of 1.0 g bone chips, with and without rinsing of the bone chips, is calculated using the regression analysis of the antibiotic solution (figure 5).

## Discussion

### Storage of antibiotics

The effects of different storage temperatures on antibiotics were studied. In addition, we examined the reaction to freezing of bone chips impregnated with antibiotics. It can be concluded that storage at -80°C and -20°C does not affect the activity of antibiotics for 1 year, and that, of bone chips impregnated with cefazolin and vancomycin up to 6 months. This shows that bone chips can be impregnated with antibiotics before storage in a bone bank. This makes surgery easier, safer and less time-consuming for the orthopaedic surgeon since they do not have to add the antibiotics during the procedure and faults can be avoided this way.

### Correlation of antibiotic quantity and zone of inhibition of bone impregnated with vancomycin and cefazolin and determination of the maximum absorbed amount

Numerous studies have concluded that morselized bone can act as a carrier for antibiotics [19-25]. These studies show that prophylactic use of bone chips impregnated with antibiotics can be effective. However, the amount of antibiotics present in the bone chips after impregnation was not determined in any of these studies, while antibiotics are effective only if their concentration is high enough to eradicate bacteria. Our study investigated the reactions, *in vitro*, of *S. epidermidis *to bone chips impregnated with cefazolin and vancomycin solutions. Correlation of antibiotic quantity and zone of inhibition of bone chips impregnated with the antibiotic solutions show that increasing amounts of cefazolin and vancomycin increase the zone of *S. epidermidis *inhibition logarithmically. At therapeutic concentrations, the correlation analysis is a good reproduction of reality, even though the zones of inhibition at high concentrations are probably not very reliable. The question can be asked whether it is necessary to use the maximum local amounts of antibiotics obtainable. It is apparent from figure 5 that increasing the antibiotic amount to above approximately 20 μg per 0.1 gram bone chips will not significantly increase the zone of inhibition. This suggests that approximately 20 μg per 0.1 gram bone chips is sufficient to eradicate the bacteria *in vitro*.

Local antibiotic therapy has to meet several other requirements in addition to that of sufficient concentration at the site of infection. Drug delivery has to be controlled in order to ensure that known adverse drug effects like deafness or renal failure are prevented [22]. Also, antibiotic concentrations should not be cytotoxic to osteoblasts or bone tissue. According to Edin, Miclau et al. [27], local levels of vancomycin of < 1000 μg/ml do not affect osteoblast replication. Cefazolin at concentrations of 200 μg/ml decreases cell replication, but does not affect the replication of osteoblasts at levels of 100 μg/ml. The concentrations of cefazolin and vancomycin solutions in our study were neither toxic to osteoblasts nor able to cause systemic side effects, but were very effective against *S. epidermidis in vitro*. However, the concentrations needed in prophylaxis against infections might be different.

To our knowledge, no studies have investigated how long high local antibiotic concentrations must be sustained to obtain effective infection prophylaxis. The prolonged release of antibiotics from antibiotic-containing bone cement did not protect against late hematogenous infections[28]. For prevention, a high local antibiotic concentration will be effective, even if this is only for a short time; one or two days, or even a few hours, can be sufficient. As long as the concentration of vancomycin and cefazolin is above the minimal inhibitory concentration (MIC) for *S. epidermidis*, it will have the maximum effect. The concentrations used in this study are well above MIC; taking into account the half-life of these antibiotics, they will stay above MIC - and be effective - for at least 8 to 10 hours. For therapeutic use, the carrier may be more effective if the antibiotic concentration is high for a longer period, preferably for 2-4 weeks. The diffusion of the antibiotic into the infected tissues takes time, as does the killing process of the bacteria.

Witso et al. [22] have shown that the elution profile from bone chips is different for different antibiotics. The elution profile of cephalosporins (cefazolin) shows a high initial release with rapid decay. After three days, all the antibiotics are eluted from the bone chips, leaving no subinhibitory amount of drug that could induce resistance. Therefore, cefazolin is a good choice for local prophylaxis. However, cephalosporins carry a small risk of hypersensitivity[29]. Vancomycin is another good choice for impregnating bone chips; it has low resistance and allergy rate and, like cefazolin, is very effective against *S. epidermidis *and *Staphylococcus. aureus*, the main pathogens colonizing biomaterials[26]. The elution profile of vancomycin is not as steep as that of cefazolin, with some elution even after seven days.

From past experience, it is likely that it will only be a matter of time before bacteria develop a mechanism of resistance against any new locally applied antibiotic. Therefore, further research into the use of combinations of antibiotics, i.e. multidrug targeting, is in order[30].

Differences between rinsing with saline and not rinsing the bone chips after impregnation were also studied. We hypothesized that rinsing the bone chips would remove antibiotic solution remaining on the surface of the chips. With cefazolin, bone chips rinsed with saline twice had a smaller free zone than bone chips that were not rinsed. This difference was not found with vancomycin. The activity of the antibiotics against *S. epidermidis *was still evident after rinsing the bone chips with saline, and high enough to eradicate the bacteria. It can therefore be concluded that the antibiotics are absorbed by the bone chips and the activity does not stem from antibiotic solution remaining on the surface of the bone chips. *In vivo *research is needed to assess the clinical value of antibiotics-impregnated bone chips.

## Conclusions

In conclusion, storage at -80°C and -20°C of antibiotic solutions, antibiotic pills and bone chips impregnated with cefazolin and vancomycin does not affect the activity of the drugs for at least one year. It is therefore possible to impregnate bone chips with antibiotics and store them in a bone bank at one of these temperatures. Also, storage of antibiotic pills at temperatures up to 37°C for 12 months does not affect their activity. However, storage of antibiotic solutions at temperatures above 20°C does affect the activity of some of the antibiotics investigated.

Based on the correlation of antibiotic quantity and zone of inhibition for cefazolin and vancomycin, optimal concentration(s) for impregnation of bone chips were determined. The correlation provides the opportunity to determine the antibiotic content of the bone chips so that a safe and reproducible bone bank product can be developed.

## Competing interests

The research study was funded by the Netherlands Bone bank Foundation.

## Authors' contributions

NMCM carried out the experiments reported here, coordinated the study and drafted the manuscript. PLCP assisted with the experiments and participated in the design and coordination of the study. PP assisted in performing the analysis, participated in the design of the study, and assisted in drafting the manuscript. BWS, PB en RMB participated in the design of the study. All authors read and approved the final manuscript.

## Pre-publication history

The pre-publication history for this paper can be accessed here:

http://www.biomedcentral.com/1471-2474/11/96/prepub

## References

[B1] Whitehouse JD Friedman ND Kirkland KB Richardson WJ Sexton DJ The impact of surgical-site infections following orthopedic surgery at a community hospital and a university hospital: adverse quality of life, excess length of stay, and extra cost Infect Control Hosp Epidemiol 2002 23 4 183 189 10.1086/502033 12002232

[B2] Plowman R The socioeconomic burden of hospital acquired infection Euro Surveill 2000 5 4 49 50 12631865 10.2807/esm.05.04.00004-en

[B3] Slooff TJ Huiskes R van Horn J Lemmens AJ Bone grafting in total hip replacement for acetabular protrusion Acta Orthop Scand 1984 55 6 593 596 10.3109/17453678408992402 6395620

[B4] Gie GA Linder L Ling RS Simon JP Slooff TJ Timperley AJ Impacted cancellous allografts and cement for revision total hip arthroplasty The Journal of bone and joint surgery 1993 75 1 14 21 10.1302/0301-620X.75B1.8421012 8421012

[B5] Schreurs BW Bolder SB Gardeniers JW Verdonschot N Slooff TJ Veth RP Acetabular revision with impacted morsellised cancellous bone grafting and a cemented cup. A 15- to 20-year follow-up The Journal of bone and joint surgery 2004 86 4 492 497 15174541

[B6] Schreurs BW Thien TM de Waal Malefijt MC Buma P Veth RP Slooff TJ Acetabular revision with impacted morselized cancellous bone graft and a cemented cup in patients with rheumatoid arthritis: three to fourteen-year follow-up J Bone Joint Surg Am 2003 85-A 4 647 652 12672840 10.2106/00004623-200304000-00010

[B7] Schreurs BW Luttjeboer J Thien TM de Waal Malefijt MC Buma P Veth RP Slooff TJ Acetabular revision with impacted morselized cancellous bone graft and a cemented cup in patients with rheumatoid arthritis. A concise follow-up, at eight to nineteen years, of a previous report J Bone Joint Surg Am 2009 91 3 646 651 10.2106/JBJS.G.01701 19255226

[B8] Schreurs BW Slooff TJ Gardeniers JW Buma P Acetabular reconstruction with bone impaction grafting and a cemented cup: 20 years' experience Clinical orthopaedics and related research 2001 393 202 215 10.1097/00003086-200112000-00023 11764350

[B9] Schreurs BW Arts JJ Verdonschot N Buma P Slooff TJ Gardeniers JW Femoral component revision with use of impaction bone-grafting and a cemented polished stem J Bone Joint Surg Am 2005 87 11 2499 2507 10.2106/JBJS.D.02547 16264127

[B10] Schreurs BW Arts JJ Verdonschot N Buma P Slooff TJ Gardeniers JW Femoral component revision with use of impaction bone-grafting and a cemented polished stem. Surgical technique J Bone Joint Surg Am 2006 2 88 Suppl 1 Pt 259 274 10.2106/JBJS.F.00340 16951098

[B11] Schreurs BW Slooff TJ Buma P Gardeniers JW Huiskes R Acetabular reconstruction with impacted morsellised cancellous bone graft and cement. A 10- to 15-year follow-up of 60 revision arthroplasties The Journal of bone and joint surgery 1998 80 3 391 395 10.1302/0301-620X.80B3.8534 9619924

[B12] Parvizi J Saleh KJ Ragland PS Pour AE Mont MA Efficacy of antibiotic-impregnated cement in total hip replacement Acta Orthop 2008 79 3 335 341 10.1080/17453670710015229 18622836

[B13] Parvizi J Pour AE Keshavarzi NR D'Apuzzo M Sharkey PF Hozack WJ Revision total hip arthroplasty in octogenarians. A case-control study J Bone Joint Surg Am 2007 89 12 2612 2618 10.2106/JBJS.F.00881 18056492

[B14] Blom AW Taylor AH Pattison G Whitehouse S Bannister GC Infection after total hip arthroplasty. The Avon experience The Journal of bone and joint surgery 2003 85 7 956 959 10.1302/0301-620X.85B7.14095 14516026

[B15] Isefuku S Joyner CJ Simpson AH Gentamicin may have an adverse effect on osteogenesis Journal of orthopaedic trauma 2003 17 3 212 216 10.1097/00005131-200303000-00010 12621263

[B16] Gristina AG Costerton JW Bacterial adherence to biomaterials and tissue. The significance of its role in clinical sepsis J Bone Joint Surg Am 1985 67 2 264 273 3881449

[B17] Belt H van de Neut D Schenk W van Horn JR Mei HC van der Busscher HJ Infection of orthopedic implants and the use of antibiotic-loaded bone cements. A review Acta Orthop Scand 2001 72 6 557 571 10.1080/000164701317268978 11817870

[B18] Winkler H Kaudela K Stoiber A Menschik F Bone grafts impregnated with antibiotics as a tool for treating infected implants in orthopedic surgery - one stage revision results Cell and tissue banking 2006 7 4 319 323 10.1007/s10561-006-9010-3 16710632

[B19] Witso E Persen L Benum P Bergh K Release of netilmicin and vancomycin from cancellous bone Acta Orthop Scand 2002 73 2 199 205 10.1080/000164702753671812 12079020

[B20] Witso E Persen L Benum P Aamodt A Husby OS Bergh K High local concentrations without systemic adverse effects after impaction of netilmicin-impregnated bone Acta Orthop Scand 2004 75 3 339 346 10.1080/00016470410001295 15260428

[B21] Winkler H Janata O Berger C Wein W Georgopoulos A In vitro release of vancomycin and tobramycin from impregnated human and bovine bone grafts The Journal of antimicrobial chemotherapy 2000 46 3 423 428 10.1093/jac/46.3.423 10980169

[B22] Witso E Persen L Loseth K Benum P Bergh K Cancellous bone as an antibiotic carrier Acta Orthop Scand 2000 71 1 80 84 10.1080/00016470052943955 10743999

[B23] Witso E Persen L Benum P Bergh K Cortical allograft as a vehicle for antibiotic delivery Acta Orthop 2005 76 4 481 486 10.1080/17453670510041457 16195062

[B24] Buttaro MA Gimenez MI Greco G Barcan L Piccaluga F High active local levels of vancomycin without nephrotoxicity released from impacted bone allografts in 20 revision hip arthroplasties Acta Orthop 2005 76 3 336 340 16156460

[B25] Buttaro MA Gonzalez Della Valle AM Pineiro L Mocetti E Morandi AA Piccaluga F Incorporation of vancomycin-supplemented bone incorporation of vancomycin-supplemented bone allografts: radiographical, histopathological and immunohistochemical study in pigs Acta Orthop Scand 2003 74 5 505 513 10.1080/00016470310017884 14620969

[B26] Montanaro L Arciola CR Baldassarri L Borsetti E Presence and expression of collagen adhesin gene (cna) and slime production in Staphylococcus aureus strains from orthopaedic prosthesis infections Biomaterials 1999 20 20 1945 1949 10.1016/S0142-9612(99)00099-X 10514072

[B27] Edin ML Miclau T Lester GE Lindsey RW Dahners LE Effect of cefazolin and vancomycin on osteoblasts in vitro Clinical orthopaedics and related research 1996 333 245 251 8981903

[B28] Malizos NK Roidis NT Karachalios TS Poultsides LA Bargiotas KA Walenkamp GHIM Management of Septic Joint Arthroplasty - The Hellenic Experience Local Antibiotics in Arthroplasty State of the art from an interdisciplinary point of view 2007 New York: Georg Thieme Verlag 109 119

[B29] Wininger DA Fass RJ Antibiotic-impregnated cement and beads for orthopedic infections Antimicrobial agents and chemotherapy 1996 40 12 2675 2679 9124821 10.1128/aac.40.12.2675 PMC163602

[B30] Murray PR Rosenthal KS Kobayashi GS Pfaller MA Medical Microbiology 1998 3 St. Louis: Mosby

